# A method for distinguishing and separating retinal microglia from infiltrating macrophages during an ocular inflammatory event

**DOI:** 10.3389/fimmu.2026.1951506

**Published:** 2026-09-22

**Authors:** Scott W. McPherson, Laura Burgstaler, Joe Sherman, Abigail Patin, Heidi Roehrich

**Affiliations:** Department of Ophthalmology and Visual Neurosciences, University of Minnesota., Minneapolis, MN, United States

**Keywords:** autoimmunity, inflammation, macrophage, microglia, retina

## Abstract

A significant challenge in the study of neuroimmune responses is the identification and separation of resident microglia from infiltrating bone marrow-derived macrophages. Therefore, we investigated whether dual labelling of Cx3cr1 and Ccr2 is sufficient to discriminate retinal microglia from infiltrating macrophages in mice that develop spontaneous autoimmune uveoretinitis–a condition dependent on infiltration of lymphocytes and antigen presenting cells from the circulation into the retina. Mice expressing red fluorescent protein (RFP) under control of the Ccr2 promoter and green fluorescent protein (GFP) under control of the Cx3cr1 promoter (Ccr2^RFP^Cx3cr1^GFP^ mice) were crossed with the uveitogenic R161H mouse line. Retinas of R161H^neg^ x Ccr2^RFP+/-^Cx3cr1^GFP+/-^ F_1_ mice, including those given an optic nerve crush injury to stimulate retinal MG expansion, and R161H^+/-^ x Ccr2^RFP+/-^Cx3cr1^GFP+/-^ F_1_ mice were analyzed for RFP and GFP expressing cells by fundoscopy, flow cytometry, fluorescence microscopy, and for Ccr2 expression by single cell RNA sequencing of retinal CD45^+^ cells. Retinas from control and optic nerve crushed R161H^neg^ x Ccr2^RFP+/-^Cx3cr1^GFP+/-^ F_1_ mice contained a single population of CD45^+^ cells expressing a uniquely high level of GFP but were devoid of RFP^+^ cells, while retinas from R161H^+/-^ x Ccr2^RFP+/-^Cx3cr1^GFP+/-^ F_1_ mice had GFP^hi^ cells and increasing amounts of GFP^neg^RFP^+^ and GFP^lo-med^RFP^+^ cells as uveoretinitis progressed. Thus, dual labelling of Ccr2 and Cx3cr1 can distinguish retinal microglia from infiltrating immune cells, and as GFP^+^, RFP^+^, and GFP^+^RFP^+^ cells can be easily separated by flow cytometry, our system provides a method for their separation for downstream cellular analysis.

## Introduction

Microglia (MG) are derived from embryonic yolk sac progenitor cells, reside within the parenchyma of central nervous system (CNS) tissue, including the retina, and are essential for the development and maintenance of neuronal tissue (1–3). MG also act as innate immune cells functioning like macrophages derived from bone marrow monocytic precursors. Under normal physiological conditions, MG are maintained in the retina with little, if any, input from bone marrow-derived precursors (4–7). However, under conditions that allow circulating myeloid cells to enter the retina, the retinal microenvironment compels those cells to become the phenotypic and functional equivalent of the endogenous MG (6, 8, 9). Thus, during ocular inflammatory events, especially those involving a breach of the blood-retina barrier, it remains a challenge to discriminate and isolate MG from infiltrating macrophages to study their role and response in the immune process.

Much effort has been spent on identifying markers that are specific for MG (7, 10–13). Tmem119 (14), a key regulator of phagocytic activity, and P2ry12 (15), essential for MG motility and migration, were found to be highly expressed in homeostatic (resting) MG and gained acceptance as unique identifiers of MG. However, recent research has shown these markers are often downregulated in MG (16–20) and can be upregulated in infiltrating myeloid cells (9, 21) during pathogenic events, calling into question their utility in discriminating MG from bone marrow-derived macrophages.

Cx3cr1 (fractalkine receptor) is expressed on a variety of circulating immune cells, however in the CNS, it is found nearly exclusively on MG (22–24). Ccr2 is a C-C chemokine receptor found on circulating myeloid cells and lymphocytes and is crucial for their migration into tissues including the CNS (25–28). Thus, we reasoned that if normal retinas lacked Ccr2^+^ cells we could use the transgenic expression of green fluorescent protein (GFP) under control of the Cx3cr1 promoter and red fluorescent protein (RFP) under control of the Ccr2 promoter to distinguish resident MG from Ccr2^+^ infiltrating immune cells including bone marrow-derived macrophages. Using a double transgenic Ccr2^RFP^Cx3cr1^GFP^ reporter mouse, we report here that normal mice and mice with a sterile inflammatory injury to the retina are replete with a unique population of GFP^hi^ cells (MG) but are devoid of RFP^+^ cells (Ccr2^+^ cells), while mice with spontaneous autoimmune uveoretinitis (SAU)--a condition dependent upon the recruitment of lymphocytes and myeloid cells into the retina--contain RFP^+^ and GFP^lo-med^RFP^+^ cells along with the unique GFP^hi^ cell population. As cells expressing these reporter genes are easily distinguished and separated by flow cytometric methods, our system provides a convenient method for isolating MG from infiltrating macrophages for further analysis.

## Methods

### Mice

Wild type B6/J mice and Ccr2^RFP^Cx3cr1^GFP^ mice (B6/J background) (29, 30) were obtained from The Jackson Laboratories (strain nos. 000664 and 032127; Bar Harbor, ME, USA). R161H mice are a CD4^+^, alpha/beta T cell receptor transgenic mouse (B10.RIII background) specific for a peptide of interphotoreceptor retinoid binding protein (IRBP, peptide 161-180) that develops clinically detectable spontaneous autoimmune uveoretinitis (SAU) starting at 3–4 weeks of age that rapidly progresses to severe disease (31–33). R161H breeders were provided by Dr. Rachel Caspi (National Eye Institute, National Institutes of Health, Bethesda, MD, USA). R161H mice were bred and used as heterozygotes (R161H^+/-^). All experimental mice were produced by mating male R161H^+/-^ mice with female B6/J or Ccr2^RFP^Cx3cr1^GFP^ mice homozygous for the reporter transgenes. The presence or absence of the transgenes was confirmed in all F_1_ mice by PCR on DNA obtained from tail snips. Heterozygosity of the R161H transgenes on a B10R.III x B6/J F_1_ background is sufficient to induce SAU (34, 35). As both Ccr2^RFP^ and Cx3cr1^GFP^ are knock-in/knock out alleles, heterozygosity of these transgenes preserve a functional allele for each gene in all cells and is sufficient for reporter gene detection. All mice were *rd8* negative (36) with all R161H^neg^ and B6/J mice showing no evidence of retinal degeneration or inflammation. All animal protocols were performed in accordance with the Association for Research in Vision and Ophthalmology (ARVO) Statement for Use of Animal in Ophthalmic and Vision Research and University of Minnesota Institutional Animal Care and Use Committee guidelines. Mice were housed under cyclic light in specific pathogen-free conditions. Euthanasia was by CO_2_ inhalation at a flow rate of 25 L/min in a multi-cage chamber with death confirmed by cervical dislocation.

### Retinal fundus imaging

Mice were anesthetized by inhalation of 3% isoflurane (Piramal, Bethlehem, PA, USA) to induce sedation then 1% isoflurane to maintain sedation using a SomnoFlo electronic vaporizer (Kent Scientific, Torrington, CT, USA). Pupils were dilated by corneal application of 4 μL of a solution containing 1 part 1.0% tropicamide (Alcon, Fort Worth, TX, USA) and 1 part 2.5% phenylephrine (SOLA Pharmaceuticals, Baton Rouge, LA, USA). A drop of 0.5% Proparacaine (Bausch + Lomb, Rochester, NY, USA) was applied after dilation but prior to fundoscopic imaging as analgesic. Corneal hydration was maintained by liberal application of Systane (Alcon) or GenTeal (Alcon). Retinal images were obtained using a Micron IV retinal imaging system (Phoenix Micron, Bend, OR, USA). White light (brightfield) and fluorescence images were obtained. A 469/35 nm band pass excitation filter in conjunction with a 525/50 nm band pass emission filter was used for GFP detection and a 562/40 nm band pass excitation filter in conjunction with a 624/40 nm band pass emission filter was used for RFP detection (Semrock, West Henrietta, NY, USA). Clinical scoring of SAU based on fundoscopy was on a scale of 0 (no disease) to 4 as described (37).

### Optic nerve crush

Mice were anesthetized with isoflurane as described above. The optic nerve was exposed as reported (38, 39) and a limited injury to the retinal ganglion cells was induced by clamping the optic nerve with Carlson DSEK graft forceps (150 μm gap, cat. no. 2197E, Ambler Surgical, Exton, PA, USA) for 3–5 seconds at a position 2 mm from the pole of the globe (40, 41). Ophthalmic antibiotic ointment (Bausch + Lomb) was then applied to the eye with daily monitoring of the eye through the end of the experiment.

### Flow cytometry

Mice were euthanized, perfused, and the retinas removed as described (38, 40, 42, 43). Retinas were individually dissociated at 37 °C for 30 minutes in a solution of 0.5 mg/mL Liberase/Blendzyme3 (Roche, Indianapolis, IN, USA) and 0.05% DNase in calcium, magnesium-free Dulbecco’s phosphate buffered saline (DPBS) with gentle trituration every 10 minutes. The retina samples were then incubated at 4 °C with the indicated fluorescent-labeled antibodies (BD Bioscience, Franklin Lakes, NJ, USA; Biolegend, San Diego, CA, USA; eBioscience, San Diego, CA, USA) for a minimum of 30 minutes, resuspended in DPBS with 2% fetal bovine serum (FBS) and analyzed using a BD Fortessa flow cytometer (BD Bioscience). An entire retina comprises a single sample, thus each sample represents the entire population of any specified immune cell(s) within that retina. Data analysis was done with FlowJo software (BD Bioscience). Blood samples were obtained by submandibular bleeding and collected in DPBS with 2 units/mL heparin (44). Red blood cells were lysed with 3 mL of 0.17 M NH_4_Cl (10 minutes at 37 °C), samples washed with DPBS, lysed again, with the remaining cells then resuspended in DPBS with 2% FBS. Samples were then stained with indicated labeled antibodies for 30–60 minutes at 4 °C, washed, and resuspended in DPBS with 2% FBS, and then analyzed. Statistical analysis of flow cytometry data given as mean or mean ± standard deviation with statistical comparison by t test as indicated.

### Fluorescence microscopy

Mice were euthanized and perfused with DPBS with 2 units/mL heparin followed by 4% paraformaldehyde in DPBS. After 5 minutes the mice were perfused again with DPBS. The eyes were removed and the retinas dissected out and cut radially to lay flat. The retinas were washed 3 x 5 minutes with DPBS, blocked for at least one hour with 10% normal donkey serum and 0.3% Triton X-100 in DPBS at room temperature and washed again 3 x 5 minutes. Primary antibodies against GFP (cat no. NB600-597, mouse anti-GFP, Novus Biologicals, Centennial, CO, USA) and RFP (cat no. NBP3-25634, rabbit anti-RFP, Novus Biologicals) were diluted 1:200 with DPBS and applied to the retinas and held at 4°C for at least 12 hours in dark conditions then washed 3 x 5 minutes with DPBS. Secondary antibodies (cat no. A21202, donkey anti-mouse Alexa Fluor 488 and cat. no. A31573, donkey anti-rabbit Alexa Fluor 647, Invitrogen/Thermo Fisher, Waltham, MA, USA) were diluted 1:800 in DPBS and applied to the retinas for 3 hours at room temperature in dark conditions. The retinas were washed 3 x 5 minutes with DPBS then stained with Hoechst 33258 (AAT Bioquest, Pleasanton, CA, USA) diluted 1:1000 with DPBS for 5 minutes at room temperature in dark conditions. The retinas were washed 2 x 5 minutes with DPBS then mounted onto slides. The retinas were imaged and analyzed using a Keyence BZ-X800 fluorescence microscope (Keyence, Itasca, IL, USA).

### Single cell RNA sequencing

Transcriptomic analysis of retinal MG is based on the methods of the Saban laboratory (45–47) and was performed in cooperation with the University of Minnesota Genomics Core facility. SAU status was assessed by fundoscopy and 6–12 retinas from each group were harvested and pooled. Groups included control mice (R161H^neg^ x B6/J F_1_) and three test groups of R161H^+/-^ x B6/J F_1_ mice — pre-SAU and with active and resolving SAU. Retinas were harvested and dissociated as described above, resuspended in Tyto running buffer, stained with anti-mouse CD45 APC (eBioscience), with CD45^+^ cells then isolated using a Miltenyi Tyto Cell Sorter (Miltenyi Biotec, Gaithersburg, MD, USA). 10,000 (control), 20,000 (pre and active SAU), and 2,000 (resolving SAU) retinal CD45^+^ cells were collected with cell count and viability (> 98.5% for all populations) determined using a LUNA-FL cell counter (Logos Biosystems, Fairfax, VA, USA). 10x Genomics (Pleasanton, CA, USA) Single Cell 3’ chemistry (v4) was used to generate Gel Bead-In Emulsions (GEM) and perform post GEM-reverse transcription cleanup, cDNA amplification, and library construction. Input RNA and cDNA quality of the libraries was assessed by Agilent D5000 TapeStation (Agilent Technologies, Santa Clara, CA, USA) and Qubit dsDNA High Sensitivity (Thermo Fisher) assays. Libraries were pooled and sequenced to target 80,000-100,000 unique reads per cell using UG 100 (Ultima Genomics, Fremont, CA, USA) sequencing. The raw sequence data was processed through Cell Ranger software (10x Genomics) to acquire.cloupe files with expression analysis performed using Loupe Browser software (10x Genomics). Identification of cell clusters was determined by the Loupe Browser software and was based on identifying highly significant (by expression p values), differentially expressed (upregulated) genes in any one cluster compared to all other clusters.

## Results

### Visual comparison of control and SAU inflamed retinas

As we reasoned that infiltrating CD45^+^ immune cells (whether macrophages or lymphocytes) could be distinguished from resident MG of the retina by expression of Ccr2, we first visualized the retinas of control (B6/J and R161H^neg^ x Ccr2^RFP+/-^Cx3cr1^GFP+/-^ F_1_ mice, n = 4 for both) and SAU (R161H^+/-^ x Ccr2^RFP+/-^Cx3cr1^GFP+/-^ F_1_ mice, n = 4) mice by fundoscopy (**Figure 1**). For all mice examined, expression of GFP under control of the Cx3cr1 promoter allowed a clear visualization of resident retinal MG in the R161Hneg x Ccr2^RFP+/-^Cx3cr1^GFP+/-^ F_1_ control mice, while retinal cells expressing Ccr2 (RFP) were not detected (**Figure 1**, second row). In contrast, in all mice that developed SAU we observed an infiltration of both RFP^+^ and GFP^+^ cells starting about 30 days of age that increased by 40 and 50 days of age (**Figure 1**, bottom three rows). That the infiltrating cells were primarily perivascular and around the optic nerve head is typically of SAU pathology (31–35), however the presence of RFP^+^ cells was observed throughout the retina.

**Figure 1:**
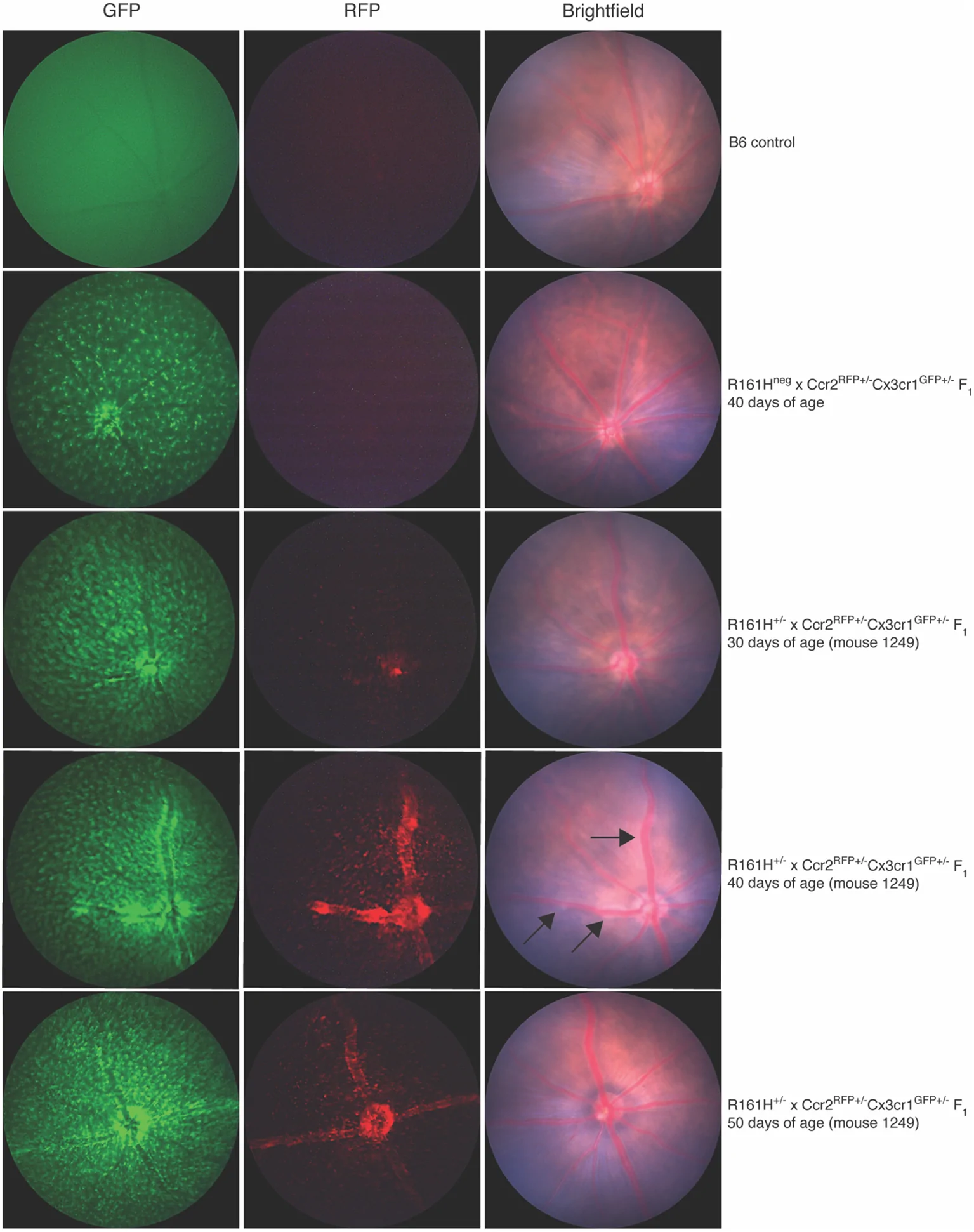
Fundoscopic examination of representative control (B6 and R161H^neg^ x Ccr2^RFP+/-^Cx3cr1^GFP+/-^ F_1_) and SAU (R161H^+/-^ x Ccr2^RFP+/-^Cx3cr1^GFP+/-^ F_1_) retinas for infiltration of RFP^+^ and RFP^+^GFP^+^ cells. Age of mouse indicated. The same field of view for each mouse/time point was analyzed by brightfield fundoscopy and for RFP and GFP fluorescence. Arrows indicate areas of perivascular infiltration at 40 days of age in the R161H^+/-^ x Ccr2^RFP+/-^Cx3cr1^GFP+/-^ F_1_ mouse (SAU score = 1.0).

Since fundoscopic analysis is limited by low sensitivity and the inability to distinguish whether the infiltrating cells were RFP^+^ only or RFP^+^GFP^+^, we analyzed the retinas of the above control and SAU mice by fluorescence microscopy at 50 days of age (**Figure 2**). In addition to analyzing the retinas of wild type B6/J mice (**Figure 2**, top), which set the background fluorescence and were negative for GFP^+^ and RFP^+^ cells, we also analyzed retinas from mice that were homozygous for the GFP and RFP reporter genes (Ccr2^RFP+/+^Cx3cr1^GFP+/+^, **Figure 2**, second row). The enhanced expression of GFP and RFP resulting from the reporter genes being homozygous allowed us to visualize the dendriform cellular morphology of the GFP^+^ cells confirming that they are resident MG and to show, even with enhanced expression of RFP, that Ccr2^+^ cells were not detected in normal retinas. As expected, analysis of R161H^neg^ x Ccr2^RFP+/-^Cx3cr1^GFP+/-^ F_1_ retinas showed GFP^+^ MG. However, these cells were negative for RFP expression nor were there any RFP^+^ only cells (**Figure 2**, third row). In contrast, R161H^+/-^ x Ccr2^RFP+/-^Cx3cr1^GFP+/-^ F_1_ retinas, even with only a modest level of SAU (score = 1.0, fundoscopy not shown) contained numerous RFP^+^ and RFP^+^GFP^+^ cells compared to the number of GFP^+^ only cells (**Figure 2**, bottom). We note that these GFP^+^ only cells have now assumed an ameboid morphology indicating that the resident MG have responded to the cellular infiltration associated with SAU.

**Figure 2:**
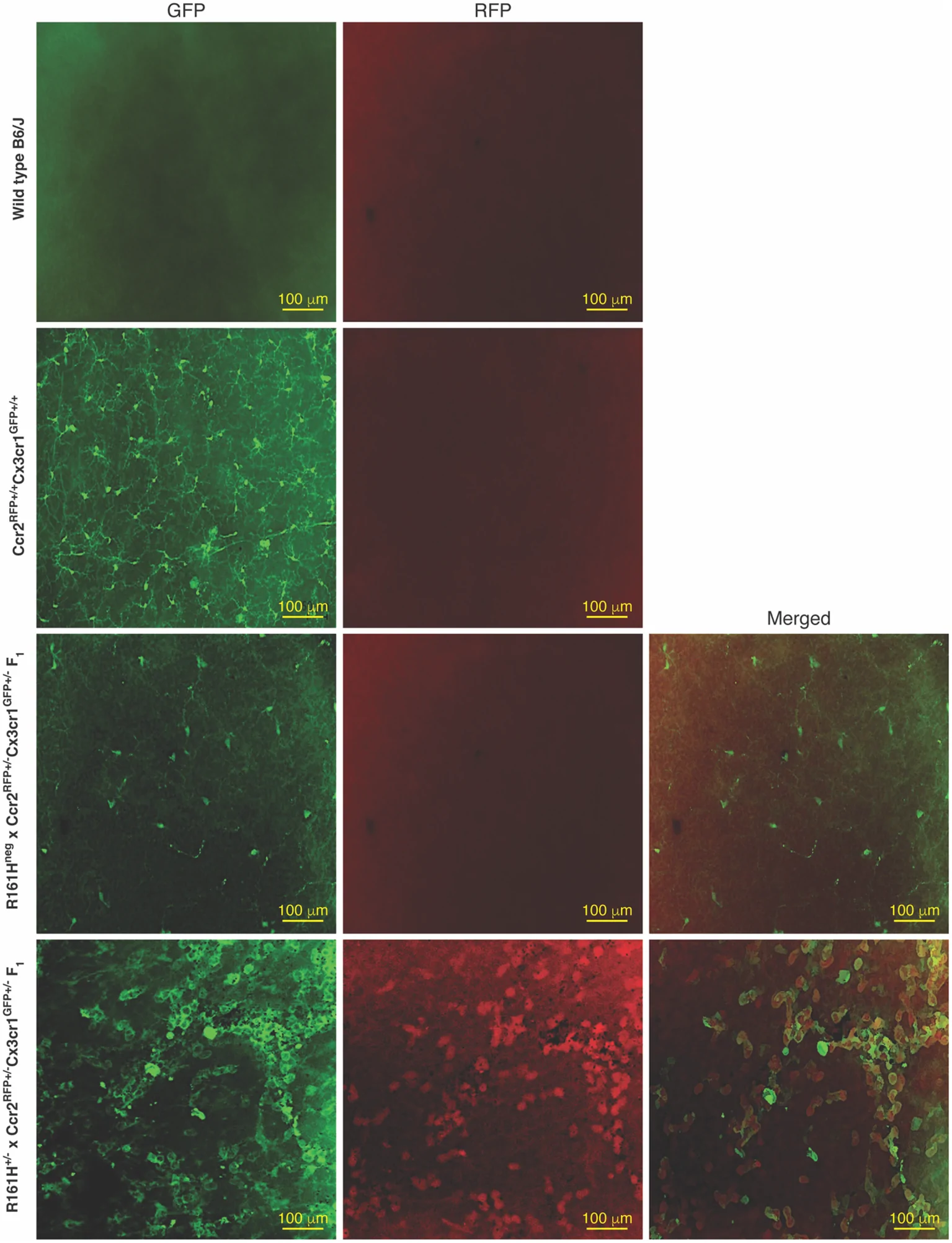
Fluorescence microscopy of representative control (B6/J, Ccr2^RFP+/+^Cx3cr1^GFP+/+^, and R161H^neg^ x Ccr2^RFP+/-^Cx3cr1^GFP+/-^ F_1_) and SAU (R161H^+/-^ x Ccr2^RFP+/-^Cx3cr1^GFP+/-^ F_1_) mouse retinas for RFP and GFP at 50 days of age. 40x magnification.

### Flow cytometry analysis of control and SAU inflamed retinas

Since fundoscopy and fluorescence microscopy only visualize a small portion of the retina, we then analyzed retinas by flow cytometry as this would allow us to investigate the entire immune cell population of the retina (**Figure 3**). As controls, we first analyzed CD45^+^ peripheral blood mononuclear cells (PBMC) from B6/J mice for background GFP and RFP fluorescence (**Figure 3A**) and then PBMC from Ccr2^RFP+/+^Cx3cr1^GFP+/+^ mice, which confirmed the presence of circulating RFP^+^, GFP^+^, and RFP^+^GFP^+^ cells (**Figure 3B**). We then analyzed all CD45^+^ cells from wild type B6/J retinas to set the background retinal RFP and GFP fluorescence (GFP^neg^ cells, **Figure 3C**). Analysis of all CD45^+^ cells from R161H^neg^ x Ccr2^RFP+/-^Cx3cr1^GFP+/-^ F_1_ retinas showed that most (82.8 ± 1.1%) of them comprised a single population that expressed a high level of GFP (Cx3cr1) suggesting they are MG (**Figure 3D**, gate 1). However, the high sensitivity of flow cytometry also allowed us to observe a very small percentage of CD45^+^RFP^+^ cells (Ccr2^+^, 1.1 ± 1.0%) that were widely scattered. As MG in physiologically normal retinas are characterized as being CD45^med^SSC^lo^ (7, 34), we specifically gated on that population (**Figure 3D**, gate 2) confirming that most of the cells (88.7 ± 4.7%) were GFP^hi^ (Cx3cr1^+^) and all were devoid of an intermediate level of GFP expression (GFP^lo-med^) and RFP (Ccr2) expression. As we and others have shown that normal retinal parenchyma does contain a small (30–50 cells), transient population of T cells (34, 48–51), we attribute the RFP^+^ (Ccr2^+^) cells found in the CD45^hi^ population as being lymphocytes. To confirm this, we analyzed the CD45^hi^ population (gate 3) by itself for RFP and found that it contained all the CD45^+^RFP^+^ cells (data not shown).

**Figure 3:**
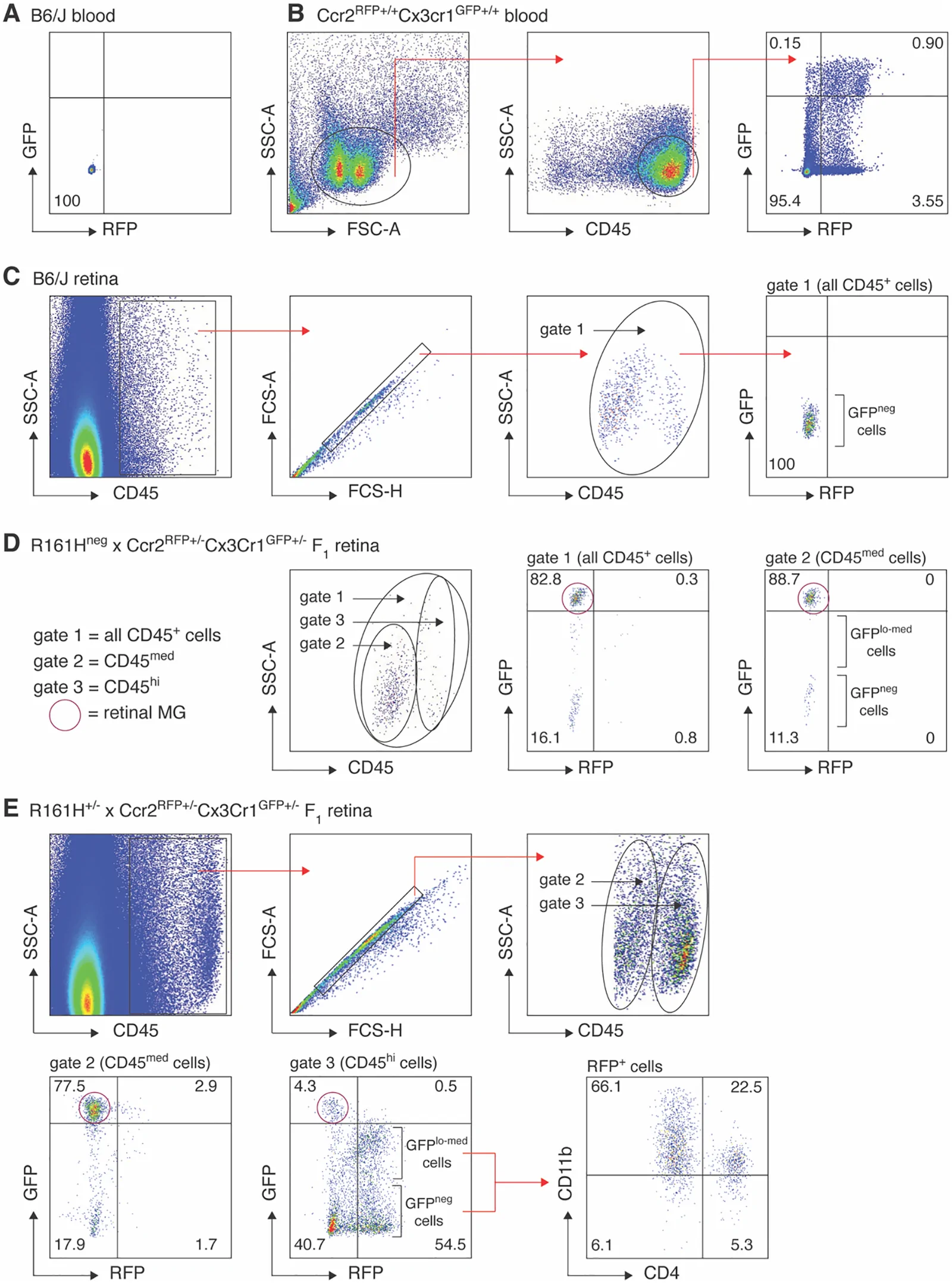
Flow cytometry distinguishes retinal MG from infiltrating macrophages. **(A, B)** Analysis of peripheral blood mononuclear cells for RFP and GFP expression showing the background levels of RFP and GFP fluorescence in B6/J mice **(A)** and the presence of RFP^+^, GFP^+^ and RFP^+^GFP^+^ cells in the circulation of Ccr2^RFP+/+^Cx3cr1^GFP+/+^ mice **(B)**. The gating strategy for selecting CD45^+^ cells from B6/J mice is the same as shown for Ccr2^RFP+/+^Cx3cr1^GFP+/+^ mice. **(C)** Representative analysis of a B6/J mouse showing background levels of RFP and GFP fluorescence in retinal CD45^+^ cells. Background fluorescence for GFP indicated as GFP^neg^ cells. **(D)** Representative analysis of R161H^neg^ x Ccr2^RFP+/-^Cx3cr1^GFP+/-^ F_1_ retinas showing that a minimal number of all CD45^+^ cells (gate 1) express Ccr2 (RFP), and that a single population comprising most of the CD45^+^ cells and specifically the CD45^med^ cells (gate 2) express a high level of Cx3cr1 (GFP). Retinal CD45^+^ cells negative for GFP expression (GFP^neg^) or with a low or intermediated level of GFP expression (GFP^lo-med^) are indicated. **(E)** Representative analysis of R161H^+/-^ x Ccr2^RFP+/-^Cx3cr1^GFP+/-^ F_1_ retinas with SAU showing a large increase in both CD45^med^ and CD45^hi^ cells with most of the RFP^+^ cells found in the CD45^hi^ population as either RFP^+^GFP^neg^ or RFP^+^GFP^lo-med^ cells and that they are myeloid cells or lymphocytes. For all analyses; retinal MG indicated by red circles, flow cytometry done at 50 days of age, n = 4 for all groups of mice, and the mean percentage of cells in each quadrant for all mice analyzed is indicated.

Consistent with our previous findings (34), R161H^+/-^ x Ccr2^RFP+/-^Cx3cr1^GFP+/-^ F_1_ retinas with SAU showed an expansion in the number of retinal MG (compare CD45^med^ populations in SSC-A *vs.* CD45 plots from **Figure 3D, E**). However, the relative percentage of MG (GFP^hi^RFP^neg^ cells) within the CD45^med^ population was slightly but statically significantly reduced (77.5% vs 88.7%, p = 0.01 by t test) in SAU due to an increase in the number of undefined GFP^neg^RFP^neg^ cells infiltrating the retina during SAU. There was an exponential increase in the number of retinal CD45^hi^ cells in SAU retinas versus controls which contained vast majority of all RFP^+^ cells (96.0 ± 3.0%, compare **Figure 3E**, gates 2 *vs.*3 plots) as either RFP^+^GFP^neg^ or RFP^+^GFP^lo-med^ cells, indicative of an infiltration of circulating lymphocytes and myeloid cells into the retina. Analysis of the RFP^+^ cells within the CD45^hi^ population for CD11b and CD4 verified they were either a myeloid cell or a T cell (**Figure 3E**, bottom right). Although most MG in R161H^+/-^ x Ccr2^RFP+/-^Cx3cr1^GFP+/-^ F_1_ retinas were found in the CD45^med^ population we did observe some associated with our defined CD45^hi^ population (**Figure 3E**, gate 3). A more stringent gating around the SSC^lo^CD45^hi^ main cell population within gate 3 eliminated the MG from this analysis (data not shown) suggesting that inflammation can alter CD45 levels in retina MG, but they retain their uniquely high level of Cx3cr1 expression. While most RFP^+^GFP^+^ cells had a lower level of GFP expression than MG from R161H^neg^ x Ccr2^RFP+/-^Cx3cr1^GFP+/-^ F_1_ control retinas (**Figure 3E**, bottom row), we observed the presence of a small number of RFP^+^ cells, particularly from the CD45^med^ population (gate 2), with the similar level of GFP expression as the resident MG. However, their lack of being a discrete population, the distinct lack of Ccr2 expression in retinal MG from R161H^neg^ x Ccr2^RFP+/-^Cx3cr1^GFP+/-^ F_1_ mice, and the lack of Ccr2 expression in MG with higher CD45 expression (**Figure 3E**, gate 3) does suggest that, aside from the small population of T cells associated with quiescent retinas, all Ccr2^+^ cells in the retinas of R161H^+/-^ x Ccr2^RFP+/-^Cx3cr1^GFP+/-^ F_1_ mice were derived from circulating precursors and, within the context of the Ccr2^RFP^Cx3cr1^GFP^ dual reporter system, that retinal MG can be identified by their uniquely high level of GFP (Cx3cr1) expression and lack of RFP (Ccr2) expression (RFP^neg^GFP^hi^ cells) in both normal and inflamed retinas.

### Expansion of retinal microglia by sterile inflammatory injury does not produce Ccr2^+^ cells within the retina

To show that expansion of retinal MG can occur without recruitment of Ccr2^+^ cells from the circulation or without the induction of Ccr2 expression in retinal cells including MG, we analyzed the retinas from six R161H^neg^ x Ccr2^RFP+/-^Cx3cr1^GFP+/-^ F_1_ mice following optic nerve crush (ONC) injury (**Figure 4**). ONC is an established and accepted model used in glaucoma research as it mimics the damage and death to retinal ganglion cells associated with increased intraocular pressure (39). In studying the retinal immune response to ONC injury we found that it is a subset of the resident retina/optic nerve microglia that can act as conventional dendritic cells that expands in response to the insult and there is no input or recruitment of circulating monocytes to the retina (38, 40, 41). As the retinal immune response to the ONC peaks 10 days post-injury (38), post-ONC analysis of the retinas by fundoscopy and flow cytometry was done at that time. Success of the ONC at inducing injury to retinal ganglion cells was evidenced by the expansion of Cx3cr1^+^ (GFP^+^) cells (MG) and their migration to form a radial pattern as they associate with the injured ganglion cells compared to pre-ONC retinas (**Figure 4A**) (38, 40, 41). However, for all mice, both pre-ONC and post-ONC retinas were devoid of Ccr2^+^ (RFP^+^) cells (**Figures 4A, B**, see also **Figure 3C**). Thus, in addition to confirming our previous findings that expansion of retinal MG in response to sterile inflammatory injury is from retinal and optic nerve progenitors (41), this data supports our idea that retinal Ccr2^+^ cells occurring during an inflammatory event represent either infiltrating macrophages or lymphoid cells and are distinct from resident MG.

**Figure 4:**
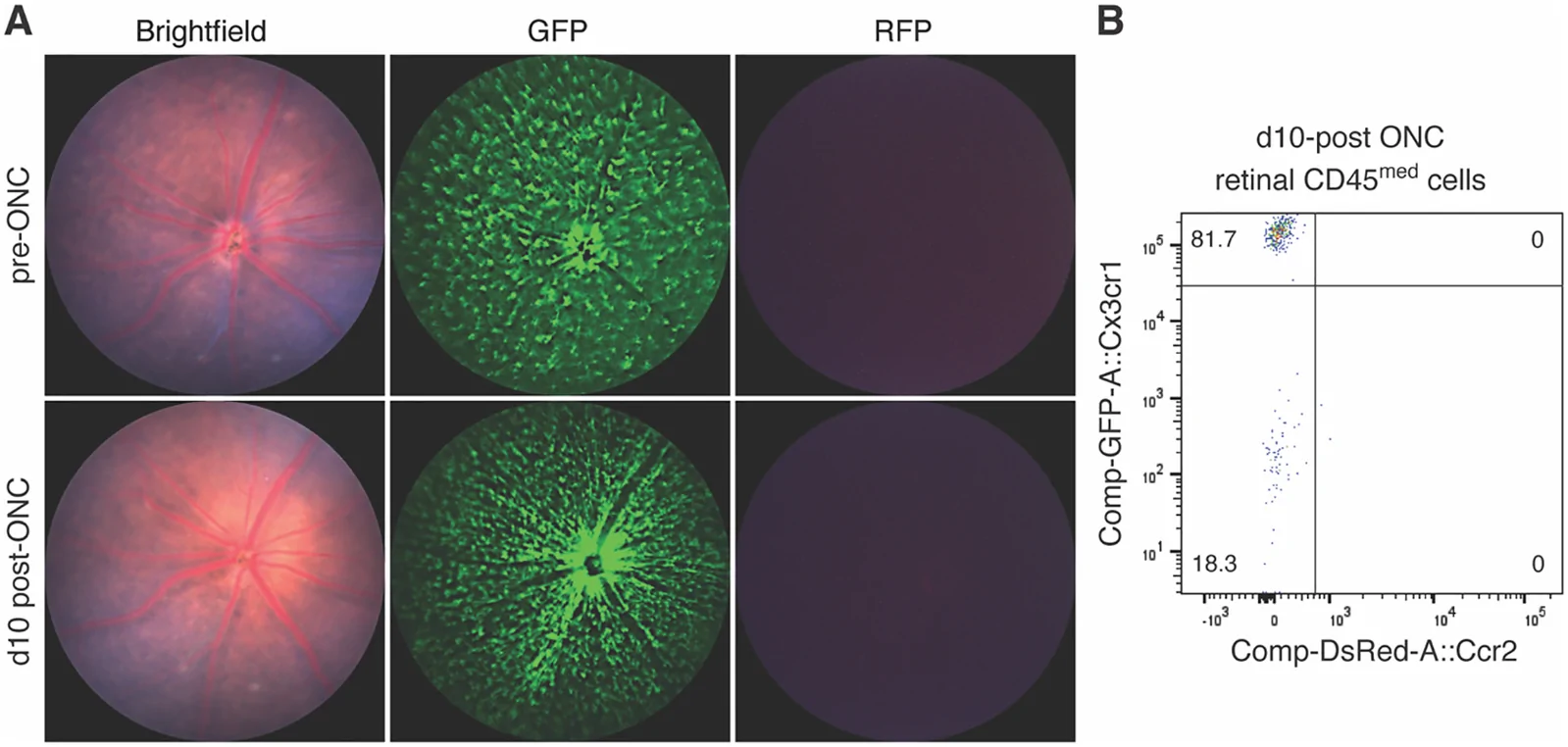
Analysis of R161H^neg^ x Ccr2^RFP+/-^Cx3cr1^GFP+/-^ F_1_ retinas for Ccr2^+^ (RFP^+^) cells following ONC. **(A)** Representative fundoscopy of pre-ONC and day 10 post-ONC retinas. **(B)** Representative flow cytometry analysis of retinal CD45^med^ cells for RFP and GFP at day 10 post-ONC with mean percentage of CD45^med^ cells expressing GFP and RFP from all examined retinas indicated.

### Single cell RNA sequencing of control and SAU inflamed retinas

As we sought further verification that Ccr2 was a valid factor in discriminating infiltrating macrophages from resident MG, we examined the data sets from an on-going single cell RNA sequencing (scRNA-seq) analysis of retinal CD45^+^ cells recovered from control (R161H^neg^ x B6/J F_1_) and SAU (R161H^+/-^ x B6/J F_1_) mice (Sherman, et. al., manuscript in preparation). Although these mice do not carry the Ccr2^RFP^ and Cx3cr1^GFP^ reporter transgenes, they do provide an orthogonal approach for assaying the distribution of endogenous Ccr2 expression in retinal CD45^+^ populations (**Figure 5**). Close examination of a small cluster of cells (cluster 1) from control mice, identified as T cells, showed Ccr2 expression in a fraction of those cells, which is consistent with the above flow cytometry data, However, Ccr2 expression was not detected in other CD45^+^ cells from control retinas. Conversely, in R161H^+/-^ x B6/J F_1_ mice, Ccr2 expression was limited and observed primarily in T cells (cluster 1) and myeloid cells (cluster 2) prior to SAU onset (pre-disease) but was found in many retinal CD45^+^ cells in mice with either active or resolving SAU. We note that the small number of Ccr2^+^ cells in pre-disease mice compared to active disease mice is consistent with our previous findings in which pre-disease mice had increased levels of retinal T cells and myeloid cells over background, but significantly fewer of those cells than was found in active disease mice (34). This data independently supports our reporter-based observations that Ccr2 expression is essentially absent from quiescent retina CD45^+^ cells, thus, in part, making it useful for distinguished retinal MG from infiltrating macrophages.

**Figure 5:**
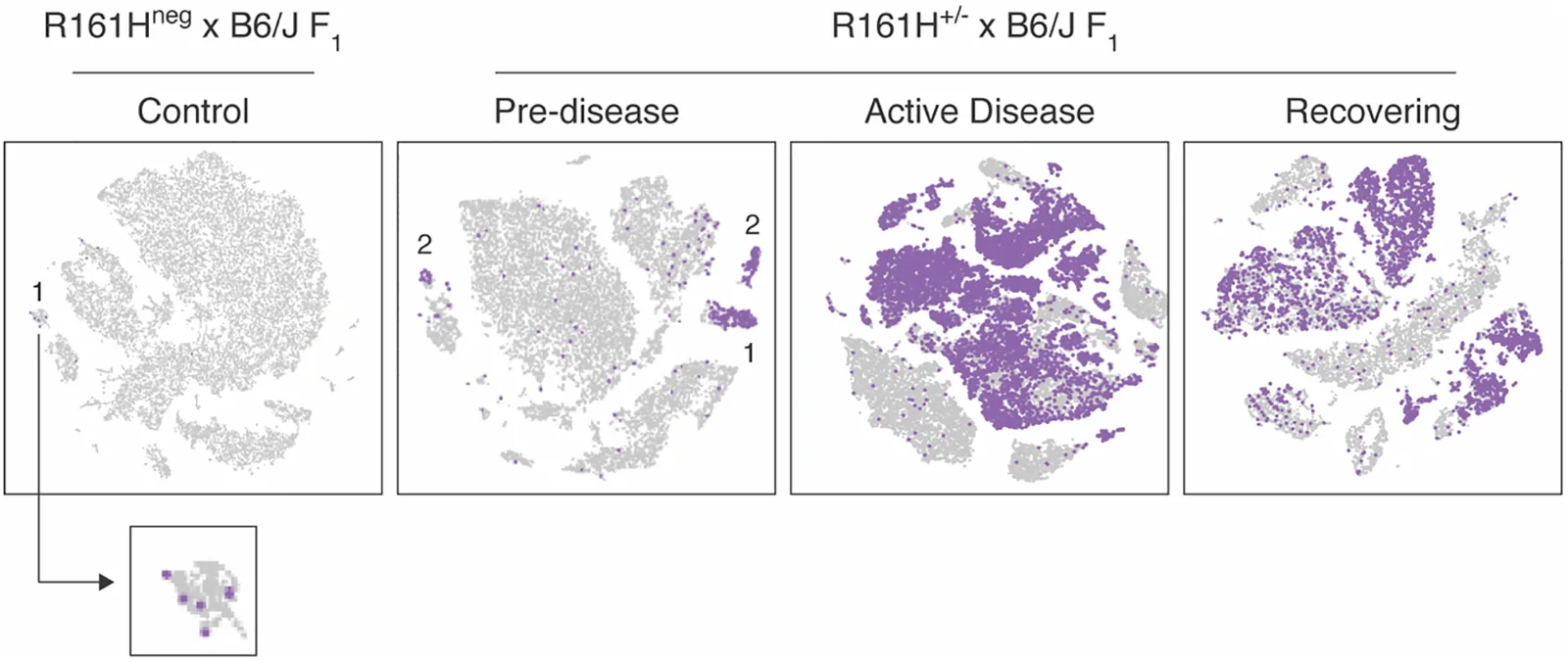
Analysis of retinal CD45^+^ cells for Ccr2 expression by scRNA-seq. Cells negative (gray) or positive (purple) for Ccr2 expression are shown. Clusters 1 (T cells) and 2 (myeloid cells) were identified by highly expressed genes. Enlargement of cluster 1 from control mice showing occasional Ccr2^+^ cell provided.

## Discussion

A clear distinction between resident MG of the retina and infiltrating macrophages and the ability to separate them is crucial for investigating their role(s) in ocular inflammatory events involving the retina. Previous protocols to identify and separate MG from infiltrating macrophages by flow cytometry have been encumbered by the high number of markers needed to identify either cell type (52, 53), the use of markers that are not necessarily unique to retinal MG [Tmem119 (10, 54),], or the use of markers that vary in their expression depending on the nature and course of the inflammatory event [MHC class II, CD11b, CD45 (55, 56),]. In this study we describe a system that relies on the dual reporting of Ccr2^RFP^ and Cx3cr1^GFP^ to uniquely separate resident MG from infiltrating macrophages in the retina.

The success of this system is based on the uniquely high expression of Cx3cr1 in MG and the near total lack of Ccr2^+^ cells in the quiescent retina. With respect to Cx3cr1^GFP^ expression, although we observed an undefined population of CD45^+^GFP^neg^ cells in the retinas of R161H^neg^ x Ccr2^RFP+/-^Cx3cr1^GFP+/-^ F_1_ mice, we did not find any CD45^+^ cells that expressed either low or intermediate levels of GFP (GFP^lo-med^). In conjunction with the idea that Cx3cr1 expression is limited to MG in CNS tissue (22–24), our finding using flow cytometry of a single GFP^+^ population in quiescent retina shows that retinal MG can, in part, be uniquely and exclusively and identified by their high level of Cx3cr1 promoted GFP expression. That Cx3cr1 expression contributes to the unique identification of retinal MG even during an inflammatory event involving infiltrating immune cells was evidenced by the continued presence of a distinct cell population with the highest level of GFP expression whether it was derived from CD45^med^ or CD45^hi^ cells in R161H^+/-^ x Ccr2^RFP+/-^Cx3cr1^GFP+/-^ F_1_ mice with active SAU. Our finding that retinal MG are characterized by a high level of Cx3cr1 expression is consistent with other studies suggesting that it is a marker of cells that have successfully established tissue residency and contribute to niche-specific immune surveillance (57–59).

It is well established that Ccr2 is necessary for the egress of monocytic cells from bone marrow and their infiltration into tissues (60, 61). While Ccr2 expression is often downregulated after tissue entry (57, 62), recent research has demonstrated there is often a continued presence of Ccr2 on infiltrating monocytes (63, 64) which can promote healing or lead to either chronic inflammation or fibrosis (65–67) depending on the local microenvironment and the persistence of Ccr2 expression. Our observation with scRNA-seq that Ccr2 is expressed on a significant portion of retinal CD45^+^ cells from mice with resolving SAU supports the idea that continued Ccr2 expression is associated with, and likely influences the ultimate outcome of, retinal inflammatory events. Persistent ocular expression of Ccr2 following experimental autoimmune uveoretinitis (EAU) has also been observed, but whether this favors chronic inflammation or promotes disease recovery is still a matter of investigation (68, 69). It is of interest to note that genetic knockouts have shown Ccr2 to be dispensable for induction of EAU in B6/J mice but required in the *Aire* knockout model of EAU (70–72).

To validate the use of Ccr2 in discriminating retinal MG from infiltrating macrophages we established by multiple techniques that Ccr2 (RFP) expression in the quiescent retina was limited to a very minute number of lymphoid cells that could be easily distinguished from MG and that stimulation of retinal MG by a method that does not involve a breach of the blood retina barrier does not promote Ccr2 expression by retinal cells. Further validation that our system uniquely identifies retinal MG as being Cx3cr1^hi^Ccr2^neg^ (GFP^hi^RFP^neg^) comes from our finding that GFP levels in RFP^+^ cells recovered from R161H^+/-^ x Ccr2^RFP+/-^Cx3cr1^GFP+/-^ F_1_ retinas at the peak of the inflammatory response remain below that found in MG from quiescent retina. Thus, using the dual Ccr2^RFP^Cx3cr1^GFP^ reporter system we can identify resident retinal MG as being Ccr2^neg^Cx3cr1^hi^ (RFP^neg^GFP^hi^) with infiltrating macrophages being either RFP^+^GFP^lo-med^ or, if Ccr2 expression has been shut down after tissue entry, GFP^lo-med^ only. As this system requires only three fluorescent antibodies or markers to identify retinal MG versus infiltrating macrophages/lymphocytes it allows the use numerous other markers to confirm the identity of the cells (*e.g.*, CD3/CD4, CD11b, Ly6G/Ly6C) and for the interrogation of cell status or function (*e.g.*, MHC-class II, CD80/86, F4/80) by flow cytometry. Further, the unique cytometric profile of retinal MG and the expression of RFP on cells infiltrating the retina allows them to be easily sorted by flow cytometry for subsequent transcriptomic analysis.

Given the variable expression and downregulation of Ccr2 after tissue entry (57, 62), the association of Cx3cr1 expression with tissue residency (59), and the influence of the retina on recruited monocytes to resemble MG as a condition for residency (6, 8, 9), it remains a formal possibility for SAU or other retinal inflammatory conditions that recruited macrophages could shut down Ccr2 expression and upregulate Cx3cr1 expression to the point where they become indistinguishable from resident MG in our reporter system. A determination of this would be difficult as it would require longitudinal study of the inflammatory condition over time with a congenic CD45 maker provided by parabiosis or bone marrow transplantation with appropriate head shielding—experiments beyond the scope of this manuscript. However, the continued expression of Ccr2 observed by us and others through late and resolving inflammation (63–67) does suggest that our system would be useful for analysis of MG and infiltrating macrophages at late stage or in chronic inflammatory conditions. More importantly, since Ccr2 expression is likely occurring in all infiltrating monocytes at early and peak inflammation, and that in our system Cx3cr1 expression in RFP^+^ cells is not as high as in endogenous MG even at peak inflammation, we believe our system is particularly useful at distinguishing and separating infiltrating macrophages from retinal MG in retinal inflammatory events from a pre-clinical stage (no obvious pathology) through early and peak inflammation. Analysis of MG versus infiltrating macrophages at the pre-clinical or early inflammatory stages could be particularly useful as many of the cellular decisions that affect the subsequent inflammation and recovery occur at these times.

Although we have begun to examine how retinal MG change and possibly influence SAU by doing scRNA-seq analysis of retinal CD45^+^ cells over the course of disease, transcriptomic analysis of retinal MG isolated from R161H^+/-^ x Ccr2^RFP+/-^Cx3cr1^GFP+/-^ F_1_ retinas will provide us with analysis precisely focused on MG. Retinal autoimmunity is unique in that it might be possible to treat using local rather systemic immune modulation that alters antigen presentation, effector T cell function, or regulatory T cell activity (73). Use of the Ccr2^RFP^Cx3cr1^GFP^ reporter system in conjunction with SAU mice (R161H^+/-^ x Ccr2^RFP+/-^Cx3cr1^GFP+/-^ F_1_ mice) will allow us to do precise mechanistic studies as to whether the immune modulation exerts its effect through resident MG, recruited antigen presenting cells, or both. In addition, years of research have led to an appreciation, albeit incompletely understood, that immune cells are chronically involved in other prevalent and debilitating retinal pathologies including age-related macular degeneration, diabetic retinopathy, and retinitis pigmentosa (74). Use of the Ccr2^RFP^Cx3cr1^GFP^ reporter system in conjunction with mouse models of these pathologies would likely be useful in understanding the contribution of retinal MG versus infiltrating macrophages, an important aspect to understanding these diseases and to the development of novel, effective treatments.

## Data Availability

The raw data supporting the conclusions of this article will be made available by the authors, without undue reservation.
